# Electronic Medical Record–Based Case Phenotyping for the Charlson Conditions: Scoping Review

**DOI:** 10.2196/23934

**Published:** 2021-02-01

**Authors:** Seungwon Lee, Chelsea Doktorchik, Elliot Asher Martin, Adam Giles D'Souza, Cathy Eastwood, Abdel Aziz Shaheen, Christopher Naugler, Joon Lee, Hude Quan

**Affiliations:** 1 Centre for Health Informatics Cumming School of Medicine University of Calgary Calgary, AB Canada; 2 Department of Community Health Sciences Cumming School of Medicine University of Calgary Calgary, AB Canada; 3 Alberta Health Services Calgary, AB Canada; 4 Data Intelligence for Health Lab Cumming School of Medicine University of Calgary Calgary, AB Canada; 5 Department of Medicine Cumming School of Medicine University of Calgary Calgary, AB Canada; 6 Department of Pathology and Laboratory Medicine Cumming School of Medicine University of Calgary Calgary, AB Canada; 7 Department of Cardiac Sciences Cumming School of Medicine University of Calgary Calgary, AB Canada

**Keywords:** electronic medical records, Charlson comorbidity, EMR phenotyping, health services research

## Abstract

**Background:**

Electronic medical records (EMRs) contain large amounts of rich clinical information. Developing EMR-based case definitions, also known as EMR phenotyping, is an active area of research that has implications for epidemiology, clinical care, and health services research.

**Objective:**

This review aims to describe and assess the present landscape of EMR-based case phenotyping for the Charlson conditions.

**Methods:**

A scoping review of EMR-based algorithms for defining the Charlson comorbidity index conditions was completed. This study covered articles published between January 2000 and April 2020, both inclusive. Embase (Excerpta Medica database) and MEDLINE (Medical Literature Analysis and Retrieval System Online) were searched using keywords developed in the following 3 domains: terms related to EMR, terms related to case finding, and disease-specific terms. The manuscript follows the Preferred Reporting Items for Systematic reviews and Meta-analyses extension for Scoping Reviews (PRISMA) guidelines.

**Results:**

A total of 274 articles representing 299 algorithms were assessed and summarized. Most studies were undertaken in the United States (181/299, 60.5%), followed by the United Kingdom (42/299, 14.0%) and Canada (15/299, 5.0%). These algorithms were mostly developed either in primary care (103/299, 34.4%) or inpatient (168/299, 56.2%) settings. Diabetes, congestive heart failure, myocardial infarction, and rheumatology had the highest number of developed algorithms. Data-driven and clinical rule–based approaches have been identified. EMR-based phenotype and algorithm development reflect the data access allowed by respective health systems, and algorithms vary in their performance.

**Conclusions:**

Recognizing similarities and differences in health systems, data collection strategies, extraction, data release protocols, and existing clinical pathways is critical to algorithm development strategies. Several strategies to assist with phenotype-based case definitions have been proposed.

## Introduction

### Background

Recent advances in computational power, increased adoption of electronic medical records (EMRs), and the subsequent rise of big data analytics in health care have opened the door to precision medicine [1]. EMRs are systemized collections of patient health information and documentation, collected in real time and stored in a digital format. EMRs were originally designed to facilitate communication in support of clinical decision-making for individual patients and to improve the quality of care. Canada and other countries have heavily promoted EMR adoption [2,3]. Globally, EMR data have been used widely for secondary purposes, such as research.

Developing case definitions, a process known as phenotyping, has become an active area of research associated with EMRs. Establishing EMR data–based phenotyping is essential for setting up the operational framework toward pursuing precision medicine, which aims to tailor medical decisions and treatments to each patient in a timely manner. EMR phenotyping allows identification and surveillance of health conditions in a timely manner and can be integrated into existing clinical flows and infrastructure. Phenotyping comorbidities using EMR data have important implications on disease management. Comorbidity is a medical condition existing simultaneously with but independently from another condition in a patient. These diseases may be related to each other by some shared association [4]. The Charlson comorbidity index [4-6] is a measure that predicts 1-year mortality based on the presence or absence of specific chronic conditions. Typically, each condition is identified through the presence of specific International Classification of Diseases (ICD) codes and assigned a score depending on the risk of death. Scores are summed for each patient to provide a total score to predict mortality [7,8]. The Charlson [5] comorbidity algorithm is the most widely used comorbidity index at present and has demonstrated the importance of classifying conditions using health data [6,7], including risk adjustment analysis, developing patient safety indicators, and identifying specific disease cohorts for research and public health applications.

### Objectives

Few reviews [9-12] have been published on developing EMR case definitions or phenotyping algorithms for selected chronic conditions, but none specifically cover all of the Charlson comorbidities. Furthermore, these articles narrowed their scope to specific perspectives [10] or specific settings (eg, inpatient or primary care only) [9,11]. These reviews report few studies utilizing natural language processing (NLP) or machine learning (ML), which emphasizes the importance of data science techniques (eg, deep learning) in the present health research. The primary objective of this study is to provide an overview of EMR-based phenotyping algorithms for the Charlson conditions. The secondary objective is to provide recommendations for health systems considering the adoption of EMR-based case phenotyping.

## Methods

### Article Screening

The methodology follows the guidelines recommended by the Preferred Reporting Items for Systematic Reviews and Meta-analysis Extension Protocols for Scoping Reviews (PRISMA-ScR) [13]. The Excerpta Medica database (Embase), and Medical Literature Analysis and Retrieval System Online (MEDLINE) databases were searched from January 2000 to April 2020 to identify peer-reviewed papers. The search strategy covered the following 3 domains: (1) terms related to EMRs, (2) terms related to case finding, and (3) disease-specific terms. We initially used validated clinical text descriptions from ICD-10 to derive search terms for selected conditions (Multimedia Appendix 1). Boolean algorithms were developed for each specific condition using the domain keywords (Multimedia Appendix 2). The cancer categories of metastatic cancer and malignant cancer were excluded, as there is already an existing review on this topic [11].

Manual screening was performed according to the following established study guidelines. Peer-reviewed journal papers were included if they were published between January 2000 and April 2020, written in English, involved human subjects and EMR, and were retrieved by the Boolean search algorithm for at least one Charlson condition. This review study focused only on case phenotyping using EMR data, and therefore, papers were excluded if they only involved administrative databases. Administrative data studies that linked EMR data were included. The presence of the Charlson conditions in each study, if reported, was defined by the presence of ICD-9 or ICD-10 codes stated in the manuscript. The full PRISMA flow diagram was created (Multimedia Appendix 3). The final search results were exported to a reference software (EndNote, Clarivate Inc) [14], and duplicates were removed.

### Characterizing the Identified Literature

A data extraction form was developed. The extracted data components included article characteristics (year and country), health care type (eg, inpatient, outpatient, and emergency), specific name of the data source, whether diagnostic codes (eg, ICD) were used, types of EMR data (eg, structured, unstructured, or imaging), techniques (eg, epidemiology/biostatistics, ML, or NLP), and whether a validation methodology was employed. The extracted data types (categorical) were recoded as binary variables to indicate whether they were employed in the algorithm. The frequencies of the algorithms, EMR settings, and countries were calculated. The identified algorithms were substratified into the following 7 types in this review based on the types of data used: (1) diagnostic codes only; (2) codes and structured data (demographics, labs, and medications); (3) diagnostic codes and free-text data; (4) diagnostic codes, structured, and free-text data; (5) structured data only; (6) free-text data only; and (7) free-text and structured data. The detailed operational definitions of case definitions used in the identified studies were also extracted. The extracted data were summarized using frequencies and graphs where applicable. STATA 14 software (StataCorp LLC) [15] was used for statistical analysis. We further summarized the used data elements, disease context, data linkage, and validation of phenotyping algorithms using the extracted tables.

## Results

### Article Screening

After 1097 duplicates were removed, a total of 3691 abstracts were identified from the electronic databases. A total of 3402 abstracts were excluded based on the title and abstract screening, resulting in 289 full-text articles for full article review. Of these, 39 articles were excluded because they did not include any Charlson conditions, and 22 articles could not be retrieved, leading to the exclusion of 61 articles. The remaining 228 articles were considered eligible for this review and analyzed. References of eligible full articles were screened, and additional articles were identified for inclusion (n=46), leading to a total of 274 articles for qualitative synthesis. Articles covering multiple disease phenotypes were counted once per phenotype, leading to a total of 299 disease phenotyping algorithms. The PRISMA diagram depicting this process is shown in Multimedia Appendix 3.

### Characteristics of the Identified Literature

**Figure 1:**
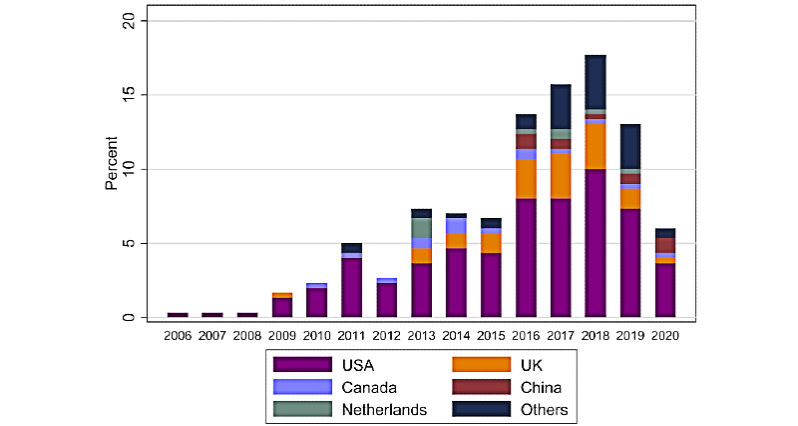
Distribution of published articles by country between January 2000 and April 2020.

**Figure 2:**
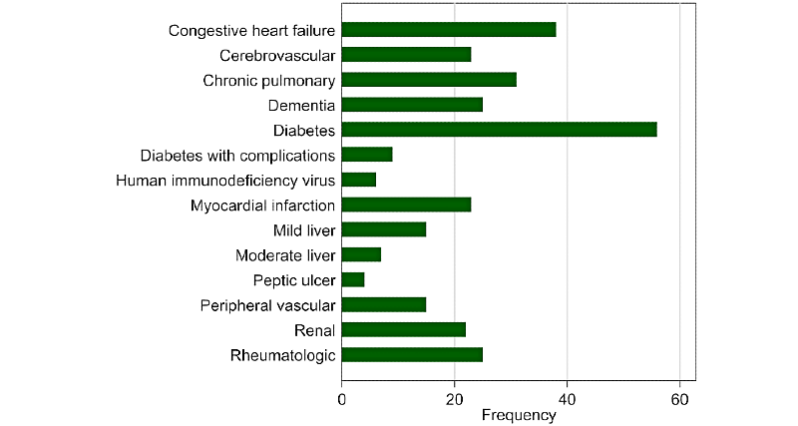
Distribution of electronic medical record data–based algorithms by Charlson disease area.

The frequencies of the algorithms, EMR settings, and countries are shown in Table 1. The complete data extraction table is presented in Multimedia Appendix 4 [16-285]. A total of 274 articles representing 299 algorithms from 22 countries were identified in this review. The majority of this work was undertaken in the United States (181/299, 60.5%), followed by the United Kingdom (42/299, 14.0%) and Canada (15/299, 5.0%). Algorithm development has steadily increased over the years, with the majority of work published after 2016. The distributions of these algorithms by the year of publication and by country are shown in Figure 1. The breakdown of the disease areas of these algorithms is shown in Figure 2.

Table 2 provides a summary of the algorithm types used for each Charlson condition. The most common algorithm types were diagnostic codes and structured data (167/299, 55.9%), followed by diagnostic codes, structured and free-text data (51/299, 17.1%), and diagnostic codes only (40/299, 13.4%). Variations in the data sources used were observed based on disease context and data availability.

These algorithms were mostly developed either in primary care (103/299, 34.4%) or inpatient (168/299, 56.2%) settings. A total of 23 algorithms (23/299, 7.7%) used data sources from inpatient and outpatient EMR. This trend was consistent across the conditions assessed in this review. The United States had the highest algorithm count across most of the assessed conditions, followed by the United Kingdom, Canada, and other nations. Detailed information about the distribution of algorithms by disease, EMR setting, and country is shown in Table 1.

We abstracted study objectives and classified different purposes for which algorithms were developed for, as well as the setting of each study (Multimedia Appendix 4). Phenotyping algorithm development was not always the primary objective for the identified studies; sometimes, it was part of a larger process. The most commonly occurring objectives of the algorithms were (1) phenotyping algorithm development (193/299, 64.5%), (2) epidemiological analysis (70/299, 23.4%), and (3) predictive modeling (19/299, 6.4%). Other objectives included designing clinical decision support and implementation tools, genome analysis, and registry development. These objectives reflect the health system delivery and clinical practice contexts in which the studies were situated.

**Table 1:** Descriptive summary of the 299 Charlson algorithms.

Disease	Algorithm count	EMR^a^ settings				Country
		Inpatient	Inpatient and outpatient	Outpatient	Other	
Myocardial infarction [16-38]	23	16	3	4	0	16 United States 2 United Kingdom 5 Others
Congestive heart failure [19,39-75]	38	22	1	14	1	27 United States 3 Sweden 8 Others
Peripheral vascular disease [19,76-89]	15	6	1	7	1	9 United States 5 United Kingdom 1 Norway
Cerebrovascular disease [19,47,57,78,83,90-107]	23	14	0	9	0	10 United States 6 United Kingdom 7 Others
Hemiplegia and paraplegia	0	0	0	0	0	0
Dementia [19,84,108-130]	25	13	1	10	1	8 United States 2 United Kingdom 1 Netherlands 1 Canada 13 Others
Chronic pulmonary disease [129,131-160]	31	14	1	16	0	16 United States 6 United Kingdom 4 Canada 5 Others
Rheumatologic disease [161-185]	25	15	1	9	0	15 United States 7 United Kingdom 3 Others
Peptic ulcer disease [186-189]	4	3	0	1	0	3 United States 1 Singapore
Diabetes [19,28,34,47,48,84,128,129,140,150,166,190-234]	56	30	6	20	0	31 United States 8 Canada 4 United Kingdom 13 Others
Diabetes, with complications [57,235-242]	9	6	1	2	0	5 United States 2 United Kingdom 1 Israel 1 China
Renal disease [47,57,243-262]	22	9	3	9	1	16 United States 2 United Kingdom 2 Spain 2 Others
Mild liver disease [189,263-276]	15	11	3	0	1	11 United States 2 China 1 Australia 1 United Kingdom
Moderate/severe liver disease [244,275-280]	7	5	0	2	0	4 United States 1 United Kingdom 1 Netherlands 1 China
HIV [137,281-285]	6	4	2	0	0	6 United States

^a^EMR: electronic medical record.

**Table 2:** The Charlson algorithm types identified in this scoping review.

Charlson condition	Algorithm type						
	Codes only	Codes and structured data	Codes and free-text data	Codes, structured, and free-text data	Structured data only	Free-text data only	Free-text and structured data
Myocardial infarction (n=23, 7.7%))	7	10	0	4	0	2	0
Congestive heart failure (n=38, 12.7%))	7	19	2	9	0	0	1
Peripheral vascular disease (n=15, 5.0%)	1	6	1	2	2	2	1
Cerebrovascular disease (n=23, 7.7%)	6	14	0	1	1	1	0
Dementia (n=25, 8.4%)	4	8	4	4	1	1	3
Chronic pulmonary disease (n=31, 10.4%)	3	17	3	3	0	4	1
Rheumatologic disease (n=25, 8.4%)	2	9	2	12	0	0	0
Peptic ulcer disease (n=4, 1.3%)	0	1	1	2	0	0	0
Diabetes (n=56, 18.7%)	6	41	1	6	1	0	1
Diabetes with complications (n=9, 3.0%)	1	8	0	0	0	0	0
Renal disease (n=22, 7.4%)	2	15	1	2	1	0	1
Mild liver disease (n=15, 5.0%)	1	11	0	2	0	0	1
Moderate/severe liver disease (n=7, 2.3%)	0	3	0	3	0	0	1
HIV (n=6, 2.0%)	0	5	0	1	0	0	0
Combined (n=299, 100.0%)	40	167	15	51	6	10	10

### Data Elements: Structured Versus Unstructured

With regard to the EMR algorithms identified in this study, structured data most commonly consisted of demographics, diagnoses, procedures, vital signs, laboratory results, and medications. Structured data elements were the most common type of data employed by clinical rule–based algorithms and included basic demographics (eg, sex and age), medications, laboratory data, and diagnostic codes. A total of 233 out of 299 (77.9%) algorithms employed key laboratory diagnostic tests based on the present clinical practice.

These structured EMR components are typically available across EMR systems. Algorithms based on diagnostic codes and structured data were used primarily (213/299, 71.2%) for chronic conditions such as diabetes, where laboratory tests and medication may be necessary and sufficient for clinical decision-making. The use of diagnostic codes depended on the EMR setting (ie, outpatient or inpatient) and the health services jurisdiction (eg, United Kingdom vs United States vs Canada) where the work took place (Multimedia Appendix 4). Types of diagnostic codes identified included ICD-9, ICD-10, Read, Oxford Medical Information System, and International Classification of Primary Care (ICPC). ICD codes were used predominantly within inpatient settings (148/168, 88.1%). These basic structured data-based definitions were enhanced by incorporating unstructured data such as free text and imaging for designing classification algorithms (Table 2) for complicated chronic conditions. In summary, the disease context determined the data elements that were used.

Unstructured free-text data (eg, discharge summaries, consult notes, and nursing notes) were incorporated in approximately 86 out of 299 (28.8%) case phenotyping algorithms. NLP techniques were used to analyze such unstructured free-text data. Many studies used controlled medical terminologies, such as the Unified Medical Language System [286] and the Systematized Nomenclature of Medicine Clinical Terms [287], in the processing of clinical notes. Both terminologies can be used by medical researchers. Many studies also employed custom vocabularies developed in consultation with clinicians or had clinicians manually annotate the free-text data to obtain the reference standard. Variations in the processing of the unstructured data were also noted. NLP processing programs such as clinical Text Analysis and Knowledge Extraction System [288], MedTagger [289], or in-house programs were employed using one of the terminologies mentioned above. This data processing converted unstructured free-text data into structured data. The converted data are often combined with existing structured data for phenotyping and disease prediction using a wide range of techniques in epidemiology, statistics, and ML. Cox regression modeling was used for survival analysis, along with incidence and prevalence in epidemiological studies. Supervised learning classification algorithms such as Naive Bayes, support vector machines, logistic regression, and neural networks are commonly used in the ML studies. The manually annotated notes or reference standard obtained from the chart review provided labels for supervised ML. 

### Disease Context

Case phenotyping algorithms exhibited 2 distinct types of approaches: clinician-derived rule-based (ie, expert-driven) and data-driven approaches. Clinician-derived rule-based approaches for defining cases were based on clinical criteria dictated by guidelines or clinical practice. These rule-based methods are generally easy to interpret and are accepted as clinically relevant. However, criteria were inconsistent within and across multiple diseases even for the clinical rule-based case phenotyping, implying that the interpretation of algorithm results may depend on choices made during the algorithm development process [290]. Despite these variations, common structured data elements were identified in each disease discipline within each context of patient care. In contrast, data-driven approaches to defining cases use information extracted from available data to determine the disease status of the patient, often with improved performance (eg, sensitivity, positive predictive value [PPV], and F1 score) compared with baseline rule-based algorithms. For example, feeding all available free-text and laboratory data for congestive heart failure (CHF) into a prediction model can classify the CHF status [73]. One study employed principal component analysis [34]. However, the association between the predictor variables and outcomes is often difficult to ascertain, and the model may be difficult to interpret.

The algorithms used various EMR data elements depending on the clinical disease context. For each disease area, unique diagnostic methods or clinical data elements were observed. Diabetes was the most commonly identified disease in our literature search (56/299, 18.7%) and will be used as an example. Case phenotyping for diabetes had fewer data element variations compared with other diseases, and algorithms involved hemoglobin A_1c_ (HbA_1c_), glucose levels, and fasting glucose as key laboratory tests and antidiabetic medications. Most diabetes algorithms did not define the severity of the disease but classified the conditions in terms of the presence or absence of type 1 or type 2 diabetes. Diabetes phenotyping studies designed patient cohort selection taking this into consideration. Developing phenotypes for identifying severe complications of diabetes did require additional data (ie, clinical narratives) and advanced methodological approaches (eg, NLP and ML), as structured data alone would not readily identify these unless diagnostic codes were included for such complications. EMR phenotypes for disease severity were sometimes developed, in the case of chronic conditions that have a widely accepted clinical severity definition. Using chronic kidney disease as an example, severity was defined according to the Kidney Disease Improving Global Outcomes [291] and the National Kidney Foundation [292] guidelines based on estimated glomerular filtration rate.

### Data Linkage

A subset of phenotyping algorithms (30/299, 10.0%) linked EMR data to disease registries or genomics data. A total of 24 out of 299 (8.0%) algorithms linked clinical and health administrative databases. All data linkage occurred in studies that used diagnostic codes. The most commonly occurring diagnostic codes were ICD-9 and ICD-10, with some regional or national diagnostic codes (eg, Read codes among UK studies). The EMR administrative data linkage context appeared mostly within primary care data-based algorithms (14/24). The UK Clinical Practice Research Datalink was linked with Hospital Episode Statistics and other administrative data to primary care EMR. The most commonly linked inpatient care data came from the Electronic Medical Records and Genomics (eMERGE) consortium [293], which provided additional validation between clinical documentation and scientific (ie, genomic) observation. These data linkage studies were employed for epidemiological analyses (improved accuracy of incidence and prevalence estimates) of diseases at the population level [83,96,212].

### Validity of Phenotyping Algorithms

Studies varied in their reporting metrics for the validity of case definition algorithms. Commonly reported metrics were sensitivity, specificity, positive predictive value, negative predictive value, accuracy, and F1 score. A total of 185 algorithms (185/299, 62.1%) employed chart review as the reference standard to calculate some of the aforementioned validation metrics. Of these 185 algorithms, 9 employed ML, 39 employed NLP, and 17 employed both ML and NLP. Of the 114 algorithms that did not conduct a chart review, 17 incorporated ML, 14 incorporated NLP, and 7 employed both ML and NLP techniques. Including free-text data as a data source in phenotyping algorithms tended to yield higher performance, with an average sensitivity of 0.906 (SD 0.110) and PPV of 0.913 (SD 0.120) when compared with studies that did not use free-text or ML (average sensitivity of 0.825 (SD 0.214) and average PPV of 0.853 (SD 0.174)). Incorporation of ML as part of the data-driven phenotyping also led to similar performance in sensitivity but weaker PPV, with an average sensitivity of 0.832 (SD 0.095) and average PPV of 0.633 (SD 0.358). In total, 59 out of 166 (35.5%) inpatient algorithms employed NLP, whereas 10 out of 93 (10.8%) primary care algorithms employed NLP. Among the works that used NLP, terminology standards were based on either Systematized Nomenclature of Medicine - Clinical Terms (SNOMED CT) or Unified Medical Language System (UMLS), although many developed their own in-house keywords. Coding standards within inpatients were based on either ICD-9 or ICD-10 depending on the timing of the study and the jurisdictions where each study took place. Similarly, primary care code standards also varied. For example, mostly Read or ICPC codes were used in the United Kingdom, whereas ICD codes were used in North America (United States and Canada). The additional data provide a specific list of ML techniques that were used in each study, if employed (Multimedia Appendix 4).

## Discussion

### EMR Phenotyping and Precision Medicine

Achieving precision medicine requires the right information to be delivered to the right personnel at the right time. Developing EMR data–based phenotypes and integrating them into existing health information systems is a pivotal step for building a learning health system. EMR phenotypes allow rapid detection of diseases and accelerate the delivery of information to clinicians who may need it to make informed clinical decisions, policymakers who may use them to obtain population information for making public health decisions, and health services organizations that may need such information for planning clinical operations or developing risk adjustment models for patient safety programs. The purposes of the case definitions identified in this review were largely achieving one of the stated objectives above.

EMR-based phenotype and algorithm development reflected the structure and data available within respective health systems. Diagnostic codes, such as ICD and present procedural terminology codes, are often used for billing purposes within inpatient and outpatient (ie, primary care) settings in certain countries (eg, the United States). These codes were also built into EMR systems (eg, problem lists). Consequently, these diagnostic codes were used extensively in algorithm development with the assumption that billing and problem list practices accurately reflect the provided care. In jurisdictional settings where ICD-based billing was not recorded directly in the EMR system during patient care (eg, inpatient care in Alberta), such assumptions could not be made and influenced the algorithm development process. Recognizing similarities and differences in data collection strategies, extraction, data release protocols, and existing clinical pathways is critical and will inform algorithm development strategies. ML and NLP techniques are increasingly being adopted in phenotyping algorithms. This is a testament to the fact that detailed records, available from free-text data, can assist with building high-performance classification algorithms.

### Data Extraction, Validity, and Quality

Developing data-driven case finding algorithms is not feasible without electronic data [294]. However, EMR data are not always easy to work with [295], as they are primarily intended to support clinical practice rather than research. EMR settings influence data collection and extraction strategies. Inpatient facilities often set up electronic data warehouses where EMR data are collected into centralized repositories, including free-text data. Primary care settings, in contrast, have variations in their systems, and studies based on primary care data often only use more common data elements such as laboratory data and demographics for multisite studies. Free-text data are less available when compared with inpatient facilities. Primary care clinics, including specialist clinics, are privately operated in many jurisdictions, whereas inpatient care may be publicly or privately operated. These different entities may not always be required to share health data or may have different data management protocols. These considerations influenced the algorithm development process, and a stark contrast in the used data elements can be observed between algorithms developed in outpatient and inpatient settings. To mitigate some of these issues, researchers conducted data linkage between data sources to expand the scope of the available data.

In addition, significant changes in the terminology and coding standards and practices in EMRs have occurred and are actively occurring. This often makes it difficult or impossible to compare or share algorithms developed for different EMR systems using different coding standards (eg, ICD-9, ICD-10, Read, SNOMED RT, SNOMED CT, and MEDCIN for diagnostic codes). Furthermore, many investigators noted that their studies were based on data from a single center, as they did not have access to external EMR data outside of their own institution. Thus, the potential lack of generalizability was a limitation for some studies. However, algorithms developed using commonly available data elements were often externally validated in multiple studies. In particular, simpler algorithms involving diagnostic codes or laboratory data appeared to be externally validated more commonly. This trend was observed in diabetes and rheumatic conditions and occurred mostly in the United States.

Variation in reported metrics (eg, sensitivity, specificity, positive predictive value, negative predictive value, area under the receiver-operator characteristic curve, and F1 score) was observed in the identified literature. Standardized metrics used in health care should be reported, including sensitivity, specificity, positive predictive value, and negative predictive value. As there is a trade-off between sensitivity and positive predictive value and both are important, it is also useful to report the F1 score, which is the harmonic mean of these 2 quantities. In addition, as class imbalance is frequently a problem in the context of disease classification, with positive instances far less common than negative instances, studies are encouraged to report metrics that account for this, such as area under the precision-recall curve [296]. At present, there are no universally accepted EMR data quality assessment metrics available, although there are various proposed data quality assessment frameworks [297]. Data quality must be assessed based on the suitability of the data to achieve a specific research objective or downstream task. We discuss this later in the recommendations.

### Limitations

This study is not without limitations. First, it is possible that our search did not encompass all qualifying articles in the field. However, our search strategy was refined and improved by systematic review search experts and librarians, and we believe our search successfully captured a broad spectrum of articles on the Charlson conditions. Second, manual screening was carried out by one individual. The objectivity of the review may have been increased by including a second reviewer. Finally, our review did not discuss methods employed for assessing EMR data quality, which depends on the context and clinical application, and is a difficult concept to measure in general. To date, there is no universally accepted data quality metric developed for EMR data, and few of the papers in this review discuss whether or how data quality was assessed in their study. Further research is required to establish the scope of practice for EMR data quality assessment.

### Recommendations on the Basis of Findings

Our review identified that case phenotyping algorithms depends on the health delivery system and disease context. We present a few observed strategies to assist with refining phenotype case definitions using the following key strategies: (1) understanding the health system structure and setting (eg, outpatient vs inpatient, coding practice) will provide a general sense of the type of EMR data that may be available; (2) considering data linkage can increase the scope of data available for algorithm development, it is important to recognize that data may not be standardized or comparable between different data sources. Additional data processing such as data recoding or data imputation may be needed; (3) identifying the relevant clinical and/or health services pathway and involving respective specialty physicians and other stakeholders as part of the algorithm development process can assist with knowledge translation; 4) employing a common data model (eg, observational medical outcomes partnership [298]) and using commonly available data elements to the possible extent can encourage widespread deployment and external validation. A common data model may differ between disease disciplines and health system areas; and (5) considering how to customize the algorithm to the needs of the end user. The needs are largely divided into clinical decision support through risk adjustment analysis, population-scale disease identification for public health initiatives, or developing methodologies to improve algorithm performance.

Health care is a unique environment, and a one-size-fits-all approach may not be appropriate. This review identified variations in EMR phenotyping, which were heavily influenced by the health care delivery setting and the disease context. To optimize performance, researchers should develop tailored algorithms that focus on the specific population of interest and the particular structure of the health system (eg, developing a primary care diabetes definition), while accounting for data issues such as variations in coding systems, clinical practice guidelines, and data quality. Once a locally developed algorithm is in place, health systems may consider implementing their case finding algorithms on standardized data models. This review identified several studies that either validated previously validated case definitions in a new setting or were refined to appropriately identify disease patients within a new setting. Having locally developed algorithms converted to standard data models will facilitate external validation and implementation, which can otherwise be a critical roadblock to the adoption of these algorithms, allowing for improved algorithm interoperability between health care systems.

The interoperability of algorithms across systems facilitates implementation within existing real-time clinical decision support systems. Easy access to developed code is also critical in validating and replicating published algorithms, after their computability has been confirmed. Analytical code and resources could be shared publicly (eg, on GitHub) to allow access for validation and implementation. The eMERGE consortium [293], CALIBER [299], and Canadian Primary Care Sentinel Surveillance Network [300], for example, have made their algorithms publicly available and have been widely adopted.

### Conclusions

We assessed EMR-based phenotyping of the Charlson conditions in health care settings. The phenotyping algorithms were locally developed and tailored to the needs and objectives of the individual studies. The health system structure and disease context determined data availability and type. The disease context dictated the common data types used for algorithm development. NLP with free-text data was employed for complex diseases that were difficult to identify with algorithms using readily available structured data. Supervised ML was employed in phenotyping algorithms, where applicable, which worked with reference standards obtained from medical chart review. Studies are encouraged to report standard health system metrics and metrics that account for class imbalance. Locally developed algorithms were validated or refined for adoption in the new setting. Locally developed disease- and setting-specific algorithms could be translated into a common data model for easier interoperability of algorithms across systems. Integrating EMR phenotyping algorithms within a health system could lead to the development of a clinical decision support system that makes use of refined existing risk adjustment scoring for risk stratification in clinical point-of-care and inform the public health and health system decision-making process, thus, leading to learning health systems.

## Appendix

Multimedia Appendix 1 Developed search terms (Medical Subject Headings) for scoping literature review.

Multimedia Appendix 2 Embase and Medical Literature Analysis and Retrieval System Online search results of Charlson terms.

Multimedia Appendix 3 Preferred Reporting Items for Systematic reviews and Meta-analyses flow diagram.

Multimedia Appendix 4 Summary spreadsheet of identified articles between January 2000 and April 2020.

## Abbreviations

CHF: congestive heart failure
eMERGE: Electronic Medical Records and Genomics
EMR: electronic medical records
ICD: International Classification of Diseases
ICPC: International Classification of Primary Care
ML: machine learning
NLP: natural language processing
PPV: positive predictive value
PRISMA-ScR: Preferred Reporting Items for Systematic reviews and Meta-analyses extension for Scoping Reviews
