# Supplementary material for: Electronic Medical Record–Based Case Phenotyping for the Charlson Conditions: Scoping Review
Source: JMIR Med Inform. 2021 Feb 1;9(2):e23934. doi: 10.2196/23934 (PMC7884219; doi:10.2196/23934)
Supplement: Multimedia Appendix 1 [file medinform_v9i2e23934_app1.docx]

### Supplementary Table 1. Developed Search Terms (MeSH) for Scoping Literature Review.

| Domain | Search Terms |
| --- | --- |
| A) Electronic medical Records | Electronic medical record or EMR or electronic medical records or EMRs or hospital information system, or HIS, electronic health records , or EHR |
|  |  |
|  |  |
| B) Case Findings | Case or identification or ascertainment or diagnosis or phenotype |
|  |  |
|  |  |
| C) Charlson Diseases |  |
| 1) Myocardial infarction | Myocardial infarction, MI, Heart attack, or acute coronary syndrome, cardiovascular |
|  |  |
| 2) Congestive heart failure | Congestive heart failure, CHF, heart failure HF, or cardiomyopathy |
|  |  |
| 3) Peripheral vascular disease | Peripheral vascular disease, PVD, peripheral arterial disease, arteriosclerosis obliterans, arterial insufficiency (of the legs), or claudication |
|  |  |
| 4) Cerebrovascular disease | cerebral vascular disease, CVD, stroke, transient cerebral ischaemic attacks, intracerebral haemorrhage, subarachnoid haemorrhage, cerebral infarction, cerebral occlusion |
|  |  |
| 5) Hemiplegia or Paraplegia | paraplegia, hemiplegia, diplegic cerebral palsy, hemiplegic cerebral palsy, plegia, paralytic syndrome, or cauda equina syndrome |
|  |  |
| 6) Dementia | Dementia, Alzheimer’s disease, or senile degeneration |
|  |  |
| 7) Chronic Pulmonary Disease | pulmonary disease, bronchitis, emphysema, COPD, Asthma, Bronchiectasis, pneumoconiosis, and pneumonitis |
|  |  |
| 8) Rheumatologic disease | rheumatoid arthritis, Felty syndrome, Still disease, rheumatoid bursitis, rheumatoid nodule, giant cell arteritis, lupus erythematosus, dermatopolymyositis, sclerosis, polymyalgia rheumatica, arthropathy |
|  |  |
| 9) Peptic ulcer disease | peptic ulcer disease, peptic ulcer, gastric ulcer, duodenal ulcer, gastrojejunal ulcer |
|  |  |
| 10) Diabetes***   - With complications, without complications | diabetes, diabetes mellitus, diabetic acidosis, diabetic coma |
|  |  |
| 11) Renal Disease | Nephritis, nephropathy, renal disease, nephrosclerosis, chronic nephritic syndrome, glomerulonephritis, chronic kidney disease, kidney failure, renal osteodystrophy, dialysis, kidney transplant |
|  |  |
| 12) Mild Liver | Chronic hepatitis, alcoholic fatty liver, alcoholic liver disease, fibrosis, cirrhosis, toxic liver disease, central haemorrhagic necrosis of liver, liver infarction, peliosis hepatis, Focal nodular hyperplasia of liver, hepatoptosis, liver transplant |
|  |  |
| 13) Moderate Liver | Oesophageal varices, gastric varices, alcoholic hepatic failure, toxic liver disease with hepatic necrosis, hepatic failure, hepatic veno-occlusive disease, portal hypertension, hepatorenal syndrome |
|  |  |
| 14) HIV | human immunodeficiency virus, HIV, acquired immunodeficiency syndrome, AIDS |
|  |  |

*** For Diabetes: after running diabetes algorithm, papers were stratified into Diabetes with complications or without complications
