# Supplementary material for: Electronic Medical Record–Based Case Phenotyping for the Charlson Conditions: Scoping Review
Source: JMIR Med Inform. 2021 Feb 1;9(2):e23934. doi: 10.2196/23934 (PMC7884219; doi:10.2196/23934)
Supplement: Multimedia Appendix 2 [file medinform_v9i2e23934_app2.docx]

Database(s): Embase 1974 to 2020 Apr 30, Ovid MEDLINE(R) 1946 to April Week 4 2020.
Search Strategy:

| **#** | **Searches** | **Results** |
| --- | --- | --- |
| 1 | CVD.mp. [mp=ti, ab, hw, tn, ot, dm, mf, dv, kw, fx, dq, nm, kf, ox, px, rx, ui, sy] | 84115 |
| 2 | Cerebrovascular disease.mp. [mp=ti, ab, hw, tn, ot, dm, mf, dv, kw, fx, dq, nm, kf, ox, px, rx, ui, sy] | 88937 |
| 3 | stroke.mp. [mp=ti, ab, hw, tn, ot, dm, mf, dv, kw, fx, dq, nm, kf, ox, px, rx, ui, sy] | 697609 |
| 4 | haemorrhage.mp. [mp=ti, ab, hw, tn, ot, dm, mf, dv, kw, fx, dq, nm, kf, ox, px, rx, ui, sy] | 81924 |
| 5 | cerebral infarction.mp. [mp=ti, ab, hw, tn, ot, dm, mf, dv, kw, fx, dq, nm, kf, ox, px, rx, ui, sy] | 50712 |
| 6 | cerebral occlusion.mp. [mp=ti, ab, hw, tn, ot, dm, mf, dv, kw, fx, dq, nm, kf, ox, px, rx, ui, sy] | 199 |
| 7 | electronic medical records.mp. [mp=ti, ab, hw, tn, ot, dm, mf, dv, kw, fx, dq, nm, kf, ox, px, rx, ui, sy] | 26152 |
| 8 | electronic health records.mp. [mp=ti, ab, hw, tn, ot, dm, mf, dv, kw, fx, dq, nm, kf, ox, px, rx, ui, sy] | 34819 |
| 9 | health information system.mp. [mp=ti, ab, hw, tn, ot, dm, mf, dv, kw, fx, dq, nm, kf, ox, px, rx, ui, sy] | 4193 |
| 10 | EMR.mp. [mp=ti, ab, hw, tn, ot, dm, mf, dv, kw, fx, dq, nm, kf, ox, px, rx, ui, sy] | 21324 |
| 11 | EHR.mp. [mp=ti, ab, hw, tn, ot, dm, mf, dv, kw, fx, dq, nm, kf, ox, px, rx, ui, sy] | 15938 |
| 12 | case.mp. [mp=ti, ab, hw, tn, ot, dm, mf, dv, kw, fx, dq, nm, kf, ox, px, rx, ui, sy] | 6992650 |
| 13 | identification.mp. [mp=ti, ab, hw, tn, ot, dm, mf, dv, kw, fx, dq, nm, kf, ox, px, rx, ui, sy] | 1583389 |
| 14 | ascertainment.mp. [mp=ti, ab, hw, tn, ot, dm, mf, dv, kw, fx, dq, nm, kf, ox, px, rx, ui, sy] | 17532 |
| 15 | diagnosis.mp. [mp=ti, ab, hw, tn, ot, dm, mf, dv, kw, fx, dq, nm, kf, ox, px, rx, ui, sy] | 8697373 |
| 16 | 1 or 2 or 3 or 4 or 5 or 6 | 927436 |
| 17 | 7 or 8 or 9 or 10 or 11 | 86822 |
| 18 | 12 or 13 or 14 or 15 | 14228914 |
| 19 | 16 and 17 and 18 | 1163 |
| 20 | heart attack.mp. [mp=ti, ab, hw, tn, ot, dm, mf, dv, kw, fx, dq, nm, kf, ox, px, rx, ui, sy] | 10339 |
| 21 | acute coronary syndrome.mp. [mp=ti, ab, hw, tn, ot, dm, mf, dv, kw, fx, dq, nm, kf, ox, px, rx, ui, sy] | 90264 |
| 22 | MI.mp. [mp=ti, ab, hw, tn, ot, dm, mf, dv, kw, fx, dq, nm, kf, ox, px, rx, ui, sy] | 128873 |
| 23 | myocardial infarction.mp. [mp=ti, ab, hw, tn, ot, dm, mf, dv, kw, fx, dq, nm, kf, ox, px, rx, ui, sy] | 510813 |
| 24 | 20 or 21 or 22 or 23 | 624895 |
| 25 | 17 and 18 and 24 | 646 |
| 26 | limit 25 to (article-in-press status or embase status or in-process status or "in data review" or in process or medline or publisher or "pubmed not medline") [Limit not valid in Ovid MEDLINE(R); records were retained] | 367 |
| 27 | limit 19 to (article-in-press status or embase status or in-process status or "in data review" or in process or medline or publisher) [Limit not valid in Ovid MEDLINE(R); records were retained] | 692 |
| 28 | congestive heart failure.mp. [mp=ti, ab, hw, tn, ot, dm, mf, dv, kw, fx, dq, nm, kf, ox, px, rx, ui, sy] | 129567 |
| 29 | heart failure.mp. [mp=ti, ab, hw, tn, ot, dm, mf, dv, kw, fx, dq, nm, kf, ox, px, rx, ui, sy] | 576456 |
| 30 | cardiomyopathy.mp. [mp=ti, ab, hw, tn, ot, dm, mf, dv, kw, fx, dq, nm, kf, ox, px, rx, ui, sy] | 222949 |
| 31 | 28 or 29 or 30 | 729880 |
| 32 | 17 and 18 and 31 | 1146 |
| 33 | limit 32 to (article-in-press status or embase status or in-process status or "in data review" or in process or medline or publisher or "pubmed not medline") [Limit not valid in Ovid MEDLINE(R); records were retained] | 628 |
| 34 | paraplegia.mp. [mp=ti, ab, hw, tn, ot, dm, mf, dv, kw, fx, dq, nm, kf, ox, px, rx, ui, sy] | 50318 |
| 35 | hemiplegia.mp. [mp=ti, ab, hw, tn, ot, dm, mf, dv, kw, fx, dq, nm, kf, ox, px, rx, ui, sy] | 34377 |
| 36 | diplegic cerebral palsy.mp. [mp=ti, ab, hw, tn, ot, dm, mf, dv, kw, fx, dq, nm, kf, ox, px, rx, ui, sy] | 679 |
| 37 | hemiplegic cerebral palsy.mp. [mp=ti, ab, hw, tn, ot, dm, mf, dv, kw, fx, dq, nm, kf, ox, px, rx, ui, sy] | 1081 |
| 38 | plegia.mp. [mp=ti, ab, hw, tn, ot, dm, mf, dv, kw, fx, dq, nm, kf, ox, px, rx, ui, sy] | 403 |
| 39 | paralytic syndrome.mp. [mp=ti, ab, hw, tn, ot, dm, mf, dv, kw, fx, dq, nm, kf, ox, px, rx, ui, sy] | 132 |
| 40 | cauda equina.mp. [mp=ti, ab, hw, tn, ot, dm, mf, dv, kw, fx, dq, nm, kf, ox, px, rx, ui, sy] | 12618 |
| 41 | 34 or 35 or 36 or 37 or 38 or 39 or 40 | 97012 |
| 42 | 17 and 18 and 41 | 23 |
| 43 | limit 42 to (article-in-press status or embase status or in-process status or "in data review" or in process or medline or publisher or "pubmed not medline") [Limit not valid in Ovid MEDLINE(R); records were retained] | 15 |
| 44 | dementia.mp. [mp=ti, ab, hw, tn, ot, dm, mf, dv, kw, fx, dq, nm, kf, ox, px, rx, ui, sy] | 307415 |
| 45 | alzheimer's disease.mp. [mp=ti, ab, hw, tn, ot, dm, mf, dv, kw, fx, dq, nm, kf, ox, px, rx, ui, sy] | 268462 |
| 46 | senile degeneration.mp. [mp=ti, ab, hw, tn, ot, dm, mf, dv, kw, fx, dq, nm, kf, ox, px, rx, ui, sy] | 104 |
| 47 | 44 or 45 or 46 | 495776 |
| 48 | 17 and 18 and 47 | 439 |
| 49 | limit 48 to (article-in-press status or embase status or in-process status or "in data review" or in process or medline or publisher or "pubmed not medline") [Limit not valid in Ovid MEDLINE(R); records were retained] | 258 |
| 50 | peripheral vascular disease.mp. [mp=ti, ab, hw, tn, ot, dm, mf, dv, kw, fx, dq, nm, kf, ox, px, rx, ui, sy] | 35284 |
| 51 | PVD.mp. [mp=ti, ab, hw, tn, ot, dm, mf, dv, kw, fx, dq, nm, kf, ox, px, rx, ui, sy] | 6338 |
| 52 | arteriosclerosis obliterans.mp. [mp=ti, ab, hw, tn, ot, dm, mf, dv, kw, fx, dq, nm, kf, ox, px, rx, ui, sy] | 5285 |
| 53 | arterial insufficiency of the legs.mp. [mp=ti, ab, hw, tn, ot, dm, mf, dv, kw, fx, dq, nm, kf, ox, px, rx, ui, sy] | 55 |
| 54 | claudication.mp. [mp=ti, ab, hw, tn, ot, dm, mf, dv, kw, fx, dq, nm, kf, ox, px, rx, ui, sy] | 32989 |
| 55 | 50 or 51 or 52 or 53 or 54 | 74371 |
| 56 | 17 and 18 and 55 | 94 |
| 57 | limit 56 to (article-in-press status or embase status or in-process status or "in data review" or in process or medline or publisher or "pubmed not medline") [Limit not valid in Ovid MEDLINE(R); records were retained] | 62 |
| 58 | pulmonary disease.mp. [mp=ti, ab, hw, tn, ot, dm, mf, dv, kw, fx, dq, nm, kf, ox, px, rx, ui, sy] | 155797 |
| 59 | bronchitis.mp. [mp=ti, ab, hw, tn, ot, dm, mf, dv, kw, fx, dq, nm, kf, ox, px, rx, ui, sy] | 78223 |
| 60 | emphysema.mp. [mp=ti, ab, hw, tn, ot, dm, mf, dv, kw, fx, dq, nm, kf, ox, px, rx, ui, sy] | 75541 |
| 61 | COPD.mp. [mp=ti, ab, hw, tn, ot, dm, mf, dv, kw, fx, dq, nm, kf, ox, px, rx, ui, sy] | 128480 |
| 62 | chronic obstructive pulmonary disorder.mp. [mp=ti, ab, hw, tn, ot, dm, mf, dv, kw, fx, dq, nm, kf, ox, px, rx, ui, sy] | 509 |
| 63 | asthma.mp. [mp=ti, ab, hw, tn, ot, dm, mf, dv, kw, fx, dq, nm, kf, ox, px, rx, ui, sy] | 446680 |
| 64 | bronchiectasis.mp. [mp=ti, ab, hw, tn, ot, dm, mf, dv, kw, fx, dq, nm, kf, ox, px, rx, ui, sy] | 32713 |
| 65 | pneumoconiosis.mp. [mp=ti, ab, hw, tn, ot, dm, mf, dv, kw, fx, dq, nm, kf, ox, px, rx, ui, sy] | 14198 |
| 66 | pneumonitis.mp. [mp=ti, ab, hw, tn, ot, dm, mf, dv, kw, fx, dq, nm, kf, ox, px, rx, ui, sy] | 38804 |
| 67 | 58 or 59 or 60 or 61 or 62 or 63 or 64 or 65 or 66 | 787962 |
| 68 | 17 and 18 and 67 | 1205 |
| 69 | limit 68 to (article-in-press status or embase status or in-process status or "in data review" or in process or medline or publisher or "pubmed not medline") [Limit not valid in Ovid MEDLINE(R); records were retained] | 663 |
| 70 | rheumatoid arthritis.mp. [mp=ti, ab, hw, tn, ot, dm, mf, dv, kw, fx, dq, nm, kf, ox, px, rx, ui, sy] | 310643 |
| 71 | Felty syndrome.mp. [mp=ti, ab, hw, tn, ot, dm, mf, dv, kw, fx, dq, nm, kf, ox, px, rx, ui, sy] | 1606 |
| 72 | Still disease.mp. [mp=ti, ab, hw, tn, ot, dm, mf, dv, kw, fx, dq, nm, kf, ox, px, rx, ui, sy] | 2819 |
| 73 | rheumatoid bursitis.mp. [mp=ti, ab, hw, tn, ot, dm, mf, dv, kw, fx, dq, nm, kf, ox, px, rx, ui, sy] | 11 |
| 74 | rheumatoid nodule.mp. [mp=ti, ab, hw, tn, ot, dm, mf, dv, kw, fx, dq, nm, kf, ox, px, rx, ui, sy] | 2640 |
| 75 | giant cell arteritis.mp. [mp=ti, ab, hw, tn, ot, dm, mf, dv, kw, fx, dq, nm, kf, ox, px, rx, ui, sy] | 16006 |
| 76 | lupus erythematosus.mp. [mp=ti, ab, hw, tn, ot, dm, mf, dv, kw, fx, dq, nm, kf, ox, px, rx, ui, sy] | 184362 |
| 77 | 70 or 71 or 72 or 73 or 74 or 75 or 76 | 485852 |
| 78 | 17 and 18 and 77 | 570 |
| 79 | limit 78 to (article-in-press status or embase status or in-process status or "in data review" or in process or medline or publisher or "pubmed not medline") [Limit not valid in Ovid MEDLINE(R); records were retained] | 243 |
| 80 | peptic ulcer disease.mp. [mp=ti, ab, hw, tn, ot, dm, mf, dv, kw, fx, dq, nm, kf, ox, px, rx, ui, sy] | 14041 |
| 81 | peptic ulcer.mp. [mp=ti, ab, hw, tn, ot, dm, mf, dv, kw, fx, dq, nm, kf, ox, px, rx, ui, sy] | 93596 |
| 82 | gastric ulcer.mp. [mp=ti, ab, hw, tn, ot, dm, mf, dv, kw, fx, dq, nm, kf, ox, px, rx, ui, sy] | 22373 |
| 83 | duodenal ulcer.mp. [mp=ti, ab, hw, tn, ot, dm, mf, dv, kw, fx, dq, nm, kf, ox, px, rx, ui, sy] | 45600 |
| 84 | gastrojejunal ulcer.mp. [mp=ti, ab, hw, tn, ot, dm, mf, dv, kw, fx, dq, nm, kf, ox, px, rx, ui, sy] | 94 |
| 85 | 80 or 81 or 82 or 83 or 84 | 136923 |
| 86 | 17 and 18 and 85 | 80 |
| 87 | limit 86 to (article-in-press status or embase status or in-process status or "in data review" or in process or medline or publisher or "pubmed not medline") [Limit not valid in Ovid MEDLINE(R); records were retained] | 41 |
| 88 | diabetes.mp. [mp=ti, ab, hw, tn, ot, dm, mf, dv, kw, fx, dq, nm, kf, ox, px, rx, ui, sy] | 1584860 |
| 89 | diabetes mellitus.mp. [mp=ti, ab, hw, tn, ot, dm, mf, dv, kw, fx, dq, nm, kf, ox, px, rx, ui, sy] | 1321518 |
| 90 | diabetic acidosis.mp. [mp=ti, ab, hw, tn, ot, dm, mf, dv, kw, fx, dq, nm, kf, ox, px, rx, ui, sy] | 387 |
| 91 | diabetic coma.mp. [mp=ti, ab, hw, tn, ot, dm, mf, dv, kw, fx, dq, nm, kf, ox, px, rx, ui, sy] | 4466 |
| 92 | 88 or 89 or 90 or 91 | 1586943 |
| 93 | 17 and 18 and 92 | 2770 |
| 94 | limit 93 to (article-in-press status or embase status or in-process status or "in data review" or medline or publisher or "pubmed not medline") [Limit not valid in Ovid MEDLINE(R); records were retained] | 1733 |
| 95 | limit 94 to yr="2000 -Current" | 1725 |
| 96 | nephritis.mp. [mp=ti, ab, hw, tn, ot, dm, mf, dv, kw, fx, dq, nm, kf, ox, px, rx, ui, sy] | 80521 |
| 97 | nephropathy.mp. [mp=ti, ab, hw, tn, ot, dm, mf, dv, kw, fx, dq, nm, kf, ox, px, rx, ui, sy] | 148337 |
| 98 | renal disease.mp. [mp=ti, ab, hw, tn, ot, dm, mf, dv, kw, fx, dq, nm, kf, ox, px, rx, ui, sy] | 154632 |
| 99 | nephrosclerosis.mp. [mp=ti, ab, hw, tn, ot, dm, mf, dv, kw, fx, dq, nm, kf, ox, px, rx, ui, sy] | 6076 |
| 100 | glomerulonephritis.mp. [mp=ti, ab, hw, tn, ot, dm, mf, dv, kw, fx, dq, nm, kf, ox, px, rx, ui, sy] | 99347 |
| 101 | chronic kidney disease.mp. [mp=ti, ab, hw, tn, ot, dm, mf, dv, kw, fx, dq, nm, kf, ox, px, rx, ui, sy] | 135427 |
| 102 | kidney failure.mp. [mp=ti, ab, hw, tn, ot, dm, mf, dv, kw, fx, dq, nm, kf, ox, px, rx, ui, sy] | 399913 |
| 103 | renal osteodystrophy.mp. [mp=ti, ab, hw, tn, ot, dm, mf, dv, kw, fx, dq, nm, kf, ox, px, rx, ui, sy] | 7384 |
| 104 | dialysis.mp. [mp=ti, ab, hw, tn, ot, dm, mf, dv, kw, fx, dq, nm, kf, ox, px, rx, ui, sy] | 331788 |
| 105 | kidney transplant.mp. [mp=ti, ab, hw, tn, ot, dm, mf, dv, kw, fx, dq, nm, kf, ox, px, rx, ui, sy] | 50474 |
| 106 | 96 or 97 or 98 or 99 or 100 or 101 or 102 or 103 or 104 or 105 | 994091 |
| 107 | 17 and 18 and 106 | 1117 |
| 108 | limit 107 to (article-in-press status or embase status or in-process status or "in data review" or medline or publisher or "pubmed not medline") [Limit not valid in Ovid MEDLINE(R); records were retained] | 596 |
| 109 | human immunodeficiency virus.mp. [mp=ti, ab, hw, tn, ot, dm, mf, dv, kw, fx, dq, nm, kf, ox, px, rx, ui, sy] | 535380 |
| 110 | Acquired Immune Deficiency Syndrome.mp. [mp=ti, ab, hw, tn, ot, dm, mf, dv, kw, fx, dq, nm, kf, ox, px, rx, ui, sy] | 142200 |
| 111 | HIV.mp. [mp=ti, ab, hw, tn, ot, dm, mf, dv, kw, fx, dq, nm, kf, ox, px, rx, ui, sy] | 728378 |
| 112 | AIDS.mp. [mp=ti, ab, hw, tn, ot, dm, mf, dv, kw, fx, dq, nm, kf, ox, px, rx, ui, sy] | 370586 |
| 113 | 109 or 110 or 111 or 112 | 1012884 |
| 114 | 17 and 18 and 113 | 848 |
| 115 | limit 114 to (article-in-press status or embase status or in-process status or "in data review" or in process or medline or publisher or "pubmed not medline") [Limit not valid in Ovid MEDLINE(R); records were retained] | 557 |
| 116 | 19 or 26 or 27 or 33 or 43 or 49 or 57 or 69 or 79 or 87 or 95 or 108 or 115 | 4809 |
