# Supplementary figures and images for: Electronic Medical Record–Based Case Phenotyping for the Charlson Conditions: Scoping Review
Source: JMIR Med Inform. 2021 Feb 1;9(2):e23934. doi: 10.2196/23934 (PMC7884219; doi:10.2196/23934)

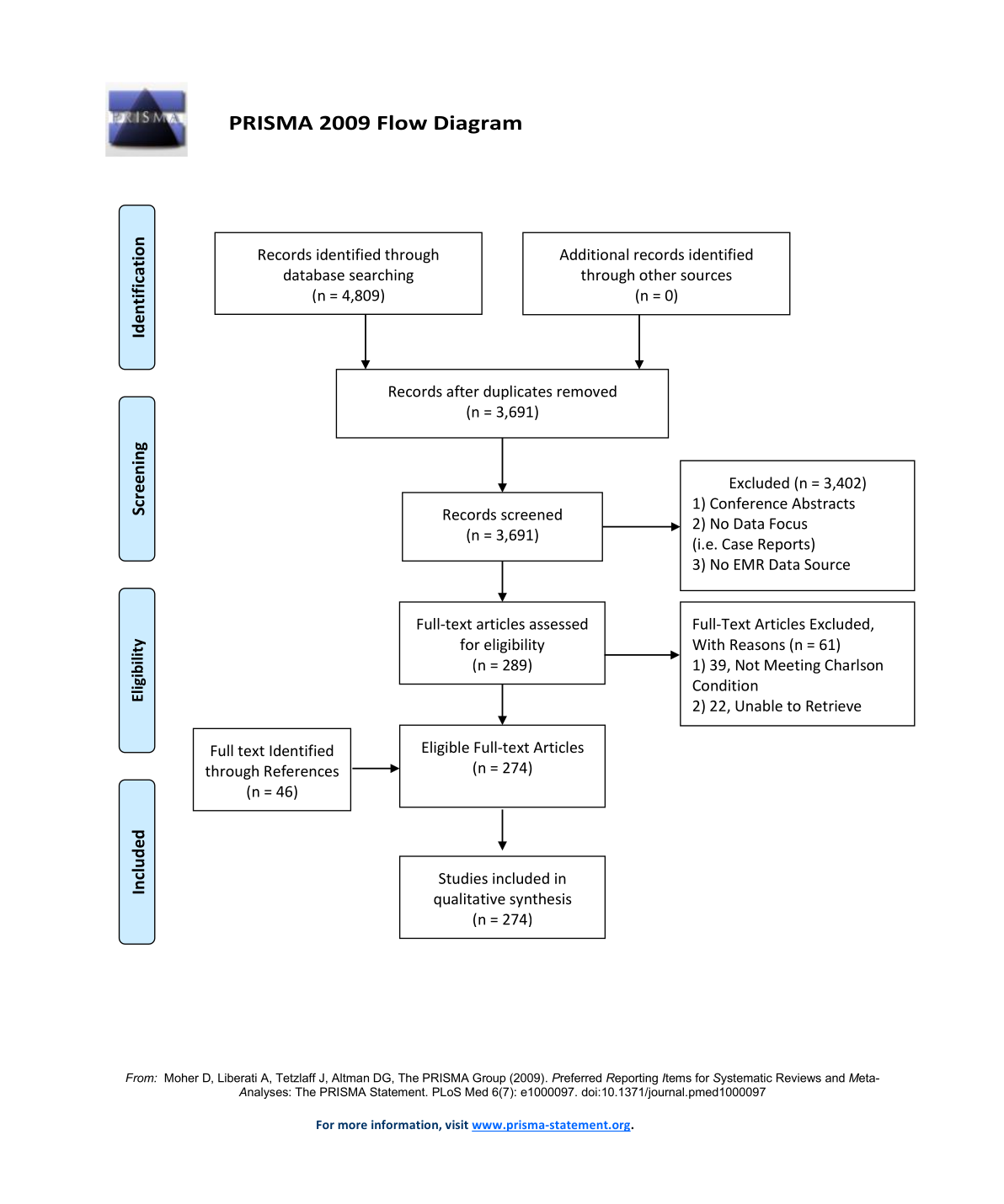

Supplement: Multimedia Appendix 3 [file medinform_v9i2e23934_app3.png]
